# Electrical neuroimaging evidence that spatial frequency-based selective attention affects V1 activity as early as 40-60 ms in humans

**DOI:** 10.1186/1471-2202-11-59

**Published:** 2010-05-06

**Authors:** Alice M Proverbio, Marzia Del Zotto, Alberto Zani

**Affiliations:** 1 Dept. of Psychology, University of Milano-Bicocca, Milan, Italy; 2 Laboratory of Experimental Neuropsychology, Neuropsychology Unit, Geneva University Hospital, Switzerland; 3 Inst. of Bioimaging and Molecular Physiology, CNR, Milano-Segrate, Italy

## Abstract

**Background:**

Karns and Knight (2009) [1] demonstrated by using ERP and gamma band oscillatory responses that intermodal attention modulates visual processing at the latency of the early phase of the C1 response (62-72 ms) thought to be generated in the primary visual cortex. However, the timing of attentional modulation of visual cortex during object-based attention remains a controversial issue.

**Results:**

In this study, EEG recording and LORETA source reconstruction were performed. A large number of subjects (29) and of trial repetitions were used (13,312). EEG was recorded from 128 scalp sites at a sampling rate of 512 Hz. Four square-wave gratings (0.75, 1.5, 3, 6 c/deg) were randomly presented in the 4 quadrants of the visual field. Participants were instructed to pay conjoined attention to a given stimulus quadrant and spatial frequency. The C1 and P1 sensory-evoked components of ERPs were quantified by measuring their mean amplitudes across time within 5 latency ranges 40-60, 60-80, 80-100, 100-120 and 120-140 ms.

**Conclusions:**

Early attention effects were found in the form of an enhanced C1 response (40-80 ms) to frequency-relevant gratings. LORETA, within its spatial resolution limits, identified the neural generators of this effect in the striate cortex (BA17), among other areas.

## Background

Neurons in the primary visual cortex (area V1) not only code simple features but also whether image elements are attended to or not [2]. However, the timing of such attentional modulation is not well understood and there is no agreement in the literature. For example, it is believed that when they are not altogether absent [3-5], attentional effects are weaker [6,7] in V1 than in associative visual areas [7-9] and occur after an additional delay. Several fMRI studies have reported robust effects of attention on V1 responses, demonstrating that attentional selection operates very early in the visual pathway [10]. According to Pessoa and coworkers [11], V1 attention effects are sometimes observed with fMRI but not with other techniques because they do not take place during the initial stimulus-related response (60-90 ms), as shown by combined fMRI/EEG and MEG studies [12], but at longer latencies in the time range 150-250 ms. According to these authors, it appears that V1 is "reactivated" in the 150-250 ms post-stimulus time range within the focus of spatial attention [8], as a sort of re-entrant feedback. In addition, some data from electrophysiological and fMRI recordings clearly indicate that spatial selection is faster and more effective than feature-based attention [13,14].

As for the timing of attentional modulation, the ERP technique is a valuable investigative tool because of its optimal temporal resolution, which, combined with modern source reconstruction techniques (such as LORETA [15]), can be informative about both the time course of attentional selection and the identification of underlying neural structures.

Indeed, while it is likely that space-based attention can modulate the extra-striate and even striate visual cortex [1,16] at a very early latency [17], numerous ERP studies have attempted to identify the stage at which attention can select non-spatial properties of the stimulus without reaching clear converging evidence. Harter and Previc [18] first attempted to assess the effects of selective attention on the activity of cortical size channels, finding an increase in amplitude of the selection negativity (SN) as early as 160 ms to attended check sizes. More recently, an ERP study with checkerboard stimuli [19] showed that the earliest signs of selective attention to check size consisted of an occipital selection negativity (OSN) at about 140 ms and a frontal selection positivity (FSP) somewhat earlier, at 120 ms. Similarly, in two studies involving selective attention to spatial frequency [20] and a given conjunction of spatial frequency and orientation [21], an attention-related anterior positivity was found at about 120 ms latency; this preceded a posterior selection negativity just after 200 ms.

A different ERP investigation of selective attention for low vs. high spatial frequency [22] showed that while attended high spatial frequency stimuli elicited an early negative difference potential (ND120) starting at about 100 ms, attended low spatial frequency stimuli elicited a positivity (PD130) in the same latency range. The neural sources of both effects were estimated by dipole modelling to lie in the extrastriate visual occipital areas, while the C1 component (60-100 ms) generated in the striate (and extrastriate) cortex was not affected by attention to spatial frequencies, according to the authors.

On the other hand, a number of other ERP studies have identified earlier P1 selective attention effects for object-based characteristics such as colour [23], colour and movement [24], check size and grating spatial frequency [25-27], transparent motion [28] and orientation [29]. Furthermore, Proverbio and Zani recently found spatial-frequency [30,31] and shape-based [32] attentional modulations of the P/N80 (C1) component as early as 60 ms, and interpreted this finding as a clear indication of striate modulation.

The possible reasons for these differences among findings and inconsistencies in the ERP literature have been widely discussed elsewhere [32] and range from large inter-individual variability in the morphology of VEPs and their attention effects, to a variety of methodological factors including differences among studies in stimulus presentation rate (e.g. too fast), inter-stimulus interval (e.g. too short: 200-500 ms in [9]; 300-550 ms in [22]), contrast and luminance levels, duration (e.g. too short: 50 ms in [9]), excessive filtering or adjar adjustments.

The overall aim of the present experiment was to investigate this matter further by using high density EEG recording from a large number of right-handed viewers engaged in a selective attention task requiring them to attend to both spatial frequencies and locations of gratings. In order to optimize the signal to noise ratio (and pick up the tiny V1 attentional modulation), large numbers of subjects (twenty-nine) and of trial repetitions were used in this study (13,312 stimulus repetitions per subject). In addition, swLORETA source reconstruction techniques were employed on the difference-waves of interest to identify the neural bases of spatial frequency-based attention effects.

It was expected that selective attention would affect early-latency responses at the level of the C1 response, a component of the VEP considered to indicate the initial afference of retinotopic regions in the human visual cortex (V1) [33], with an onset over the central parieto-occipital scalp between 45 and 60 ms. A similar outcome was recently found for the attentional modulation of Gabor pattern luminance [16].

## Methods

### Participants

Twenty-nine university students (13 males and 16 females) ranging in age from 20 to 30 years; mean age = 23.23 years) took part in this experiment as volunteers. All participants had a normal or corrected-to-normal vision with right eye dominance. They were strictly right-handed as assessed by the Edinburgh Inventory and none of them had any left-handed relatives. Experiments were conducted with the understanding and written consent of each participant according to the Declaration of Helsinki (BMJ 1991; 302: 1194) with approval from the Ethical Committee of the Italian National Research Council (CNR) and in compliance with APA ethical standards for the treatment of human volunteers (1992, American Psychological Association). Subjects gained academic credits for their participation. The data of three subjects were subsequently discarded because of excessive eye-movements.

### Stimuli and Procedure

Participants were seated in a dimly lit, electrically shielded cubicle and gazed binocularly on a fixation point permanently present in the centre of a visual display situated 114 cm in front of them. They were instructed to avoid any kind of eye or body movement. Four square-wave luminance-modulated vertical gratings were used as stimuli. Gratings produced stimulation at 0.75, 1.5, 3, 6 c/deg visual angle. Contrast was 40% and presentation duration was 80 ms. The patterns (3.5° high × 5° wide) were replaced by an isoluminant grey field (35 cd/m^2^) for a randomly varying interval between 690 and 790 ms (SOA 770-870 ms). Stimulus and background had equal average luminance to avoid flash stimulation. Mean grating luminance (on average 43 cd/m^2^) was measured for each spatial frequency and space location using a Minolta CS-100 photometer. An ANOVA performed on luminance values showed no significant difference between stimuli, thus proving stimulus and background equiluminance.

The gratings were randomly presented in pattern-onset mode within the left and right upper and lower hemifields of a PC screen. Within each quadrant, grating stimulation began 2.5° above or below the horizontal meridian, and 1.5° lateral to the vertical meridian, and extended to 3.5° above or below the horizontal meridian and 5° along it.

Different conjoined selective attention conditions were administered in random order for 0.75 or 6 c/deg within each quadrant to each subject. Irrespective of target frequency, gratings of 1.5 and 3 c/deg always served as potential distracters. Before the beginning of each task condition, participants were instructed to pay conjoined attention to a spatial frequency within a given quadrant of visual space (e.g. 6 c/deg in the right upper field) and to ignore the other combinations of frequencies and quadrants. Thus, although the physical stimuli remained unchanged, attention shifted across spatial frequency and space location. In this way, the same stimulus in different attention conjunction conditions could be: (i) relevant in both spatial location and spatial frequency (L+F+); (ii) relevant in spatial location but irrelevant in spatial frequency (L+F-); (iii) irrelevant in spatial location but relevant in spatial frequency (L-F+); or (iv) irrelevant in both features (L-F-).

To monitor spatial and stimulus attention selectivity, the volunteers were instructed to press a button to targets as accurately and quickly as possible, allowing their reaction time (RT) to be recorded as well. For each stimulus target and quadrant, eight blocks of 208 trials were replicated. During a single block of trials, each of the four gratings (i.e. one target and three "distracters") was equiprobably presented 13 times within the four quadrants in a completely random sequence. Trial order changed randomly from block to block. In half the blocks, the participants pushed the detection-RT button with the index finger of the left hand, whereas in the other half they used the right hand. The order of hands was counterbalanced across participants. The order with which attention tasks were administered and spatial locations attended was counterbalanced across participants and experimental sessions. For each of the eight conjoined attention conditions, eight different blocks of trials were run for a total of 64 blocks per subject. Overall, the global time of EEG recording was about 6 hours (with a two-minute pause after each block and longer coffee/candy breaks every 10 blocks), so the experiment took place over two consecutive days. For each individual subject, 13,312 stimuli were presented and as many EEG epochs were obtained.

### EEG recording and analysis

**Figure 1:**
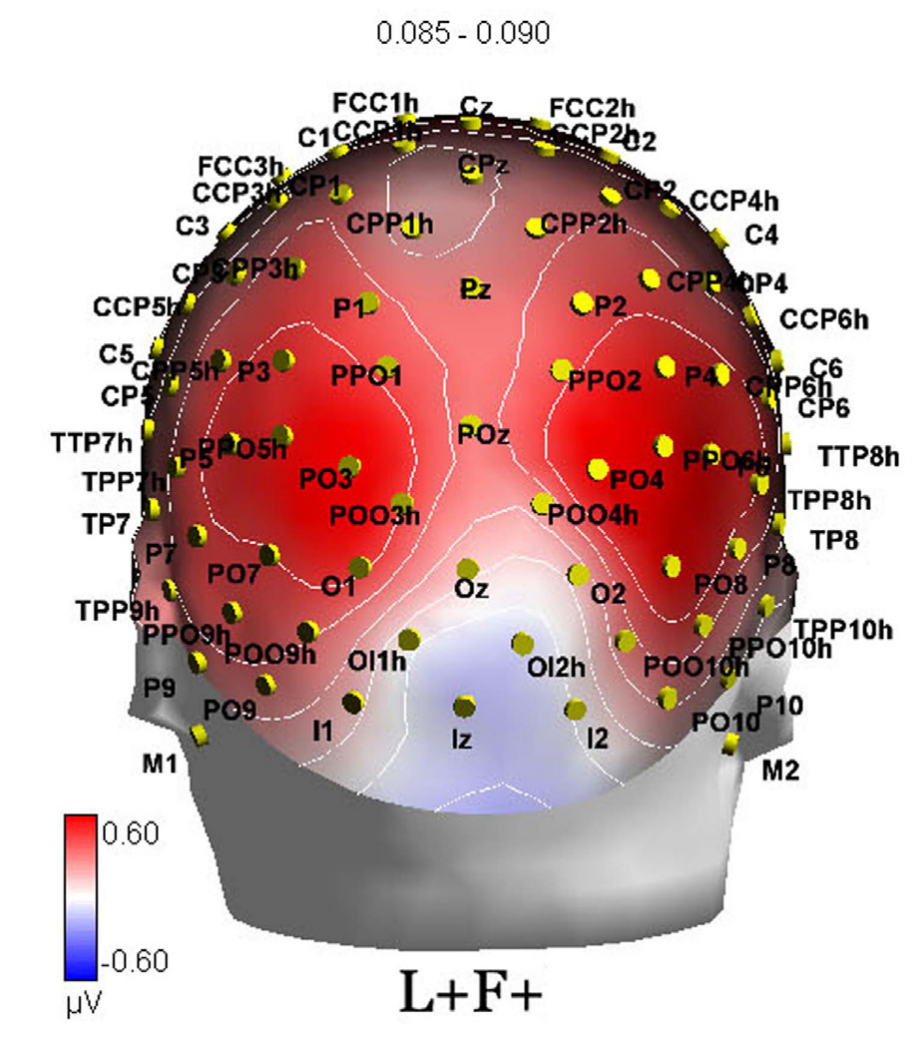
**Posterior view of electrode scalp sites (topographic map of brain activity to targets (85-90 ms)**.

The EEG was continuously recorded from 128 sites at a sampling rate of 512 Hz (see Fig. 1 for a posterior view of electrode locations). Vertical eye movements were recorded by two electrodes placed below and above the right eye, while horizontal movements were recorded from electrodes placed at the outer canthi of the eyes. Linked ears served as the reference lead. The EEG and electro-oculogram (EOG) were amplified with a half-amplitude band pass of 0.016-100 Hz. Electrode impedance was kept below 5 kΩ. EEG epochs were synchronized with the onset of stimulus presentation and analyzed by ANT-EEProbe software. Computerized artefact rejection was performed before averaging to discard epochs in which eye movements, blinks, excessive muscle potentials or amplifier blocking occurred. EEG epochs associated with an incorrect behavioural response were also excluded. The artefact rejection criterion was a peak-to-peak amplitude exceeding 70 μV, and the rejection rate was ~5%. ERPs were averaged offline from -200 ms before to 800 ms after stimulus onset and were low-pass filtered up to 50 Hz offline. ERP components were identified and measured with reference to the average baseline voltage over the interval -100 ms to 0 ms relative to stimulus onset.

Topographical voltage maps of ERPs were made by plotting colour-coded isopotentials derived by interpolating voltage values between scalp electrodes at specific latencies. Low Resolution Electromagnetic Tomography (LORETA [15]) was performed on ERP difference waves at various time latencies. LORETA, which is a discrete linear solution to the inverse EEG problem, corresponds to the 3D distribution of electric neuronal activity that has maximum similarity (i.e. maximum synchronization), in terms of orientation and strength, between neighbouring neuronal populations (represented by adjacent voxels).

A realistic boundary element model (BEM) was derived from a T1 weighted 3D MRI data set by segmentation of the brain tissue. The BEM model consisted of one homogenic compartment made up of 3446 vertices and 6888 triangles. The head model was used for intra-cranial localization of surface potentials. Segmentation and head model generation were performed using the ASA package [34]. In this study an improved version of standardized low-resolution brain electromagnetic tomography (sLORETA) was used, which incorporates a singular value decomposition-based lead field weighting: (swLORETA)[35]. swLORETA is complemented by equivalent dipole modelling. The electromagnetic dipoles are shown as arrows and indicate the position, orientation and magnitude of the dipole modelling solution applied to the ERP difference wave in the specific time window. Source space properties were: grid spacing = 5 mm; estimated SNR = 3.

Average ERPs were obtained separately for each electrode site, spatial frequency, spatial location and attention conjunction condition. Only the ERPs to the lowest (i.e. 0.75 cpd) and highest (i.e. 6 cpd) spatial frequency were analyzed under the different attention conditions, since selective attention had to be paid uniquely to those frequencies, whereas the intermediate frequencies (i.e. 1.5 and 3 cpd) always had to be ignored by the subjects.

**Figure 2:**
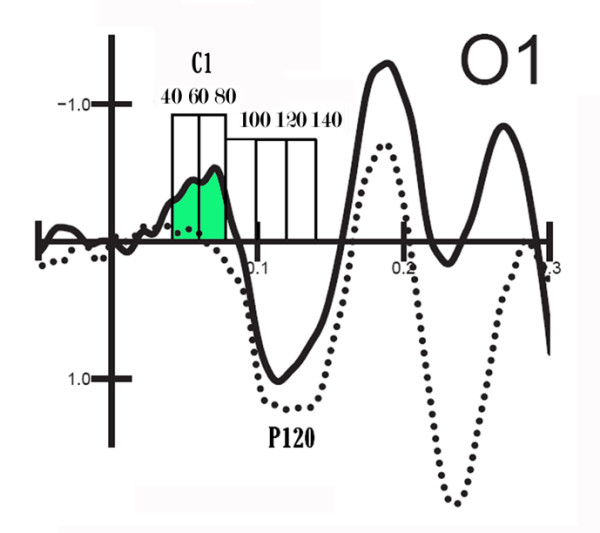
**Enlargement of time scale to show the five time windows use to mean quantified C1 and P1 amplitude values**. VEPs to 6 c/d gratings when relevant or irrelevant in spatial frequency.

Since the main goal of the present study was to investigate the possible attention modulation of early sensory processing, the results reported here mainly concern the effects of attention on the amplitude of the earliest (C1) and following (P1) sensory-evoked components elicited by stimulus gratings at occipital leads. These sensory components were analyzed by automatically measuring their mean amplitudes across time within the five latency ranges 40-60, 60-80, 80-100, 100-120 and 120-140 ms post-stimulus (see Fig. 2).

Separate six-way repeated-measure analyses of variance (ANOVAs) were performed on the mean values computed in the various time windows. Factors were: grating spatial frequency (0.75 and 6 c/deg), quadrant of visual field (upper left and right, lower left and right), location relevance (L+ = location relevant, L- = location irrelevant), frequency relevance (F+ = frequency relevant, F- = frequency irrelevant), electrode site (mesial occipitals: O1, O2; POO3 h POO4 h and lateral/occipitals PO3, PO4; PO7, PO8), and cerebral hemisphere (right and left).

For each participant, reaction times exceeding mean ± 2 standard deviations were excluded. Behavioural data underwent a four-way ANOVA whose factors of variability were: spatial frequency (0.75, 6 c/deg), hemifield (RVF, LVF), hemispace (upper, lower), hand of response (left, right). Possible type 1 errors associated with inhomogeneity of variance were controlled by the Greenhouse-Geisser procedure.

Post-hoc Tukey tests were used for multiple comparisons of means.

## Results

### Behavioral data

RTs were faster to 6 (507 ms) than 0.75 c/deg (513) gratings (F[1,25] = 3.943, p = 0.05). The interaction of spatial frequency × hemispace (F[1,25] = 21.591, p < 0.0001) indicated a frequency effect only for upper quadrants, with faster RTs to 6 (504 ms) rather than to 0. 75 c/deg gratings (515 ms) Accuracy data did not lead to any statistical significance (mean omission rate was 8.665%).

### Electrophysiological data

**Figure 3:**
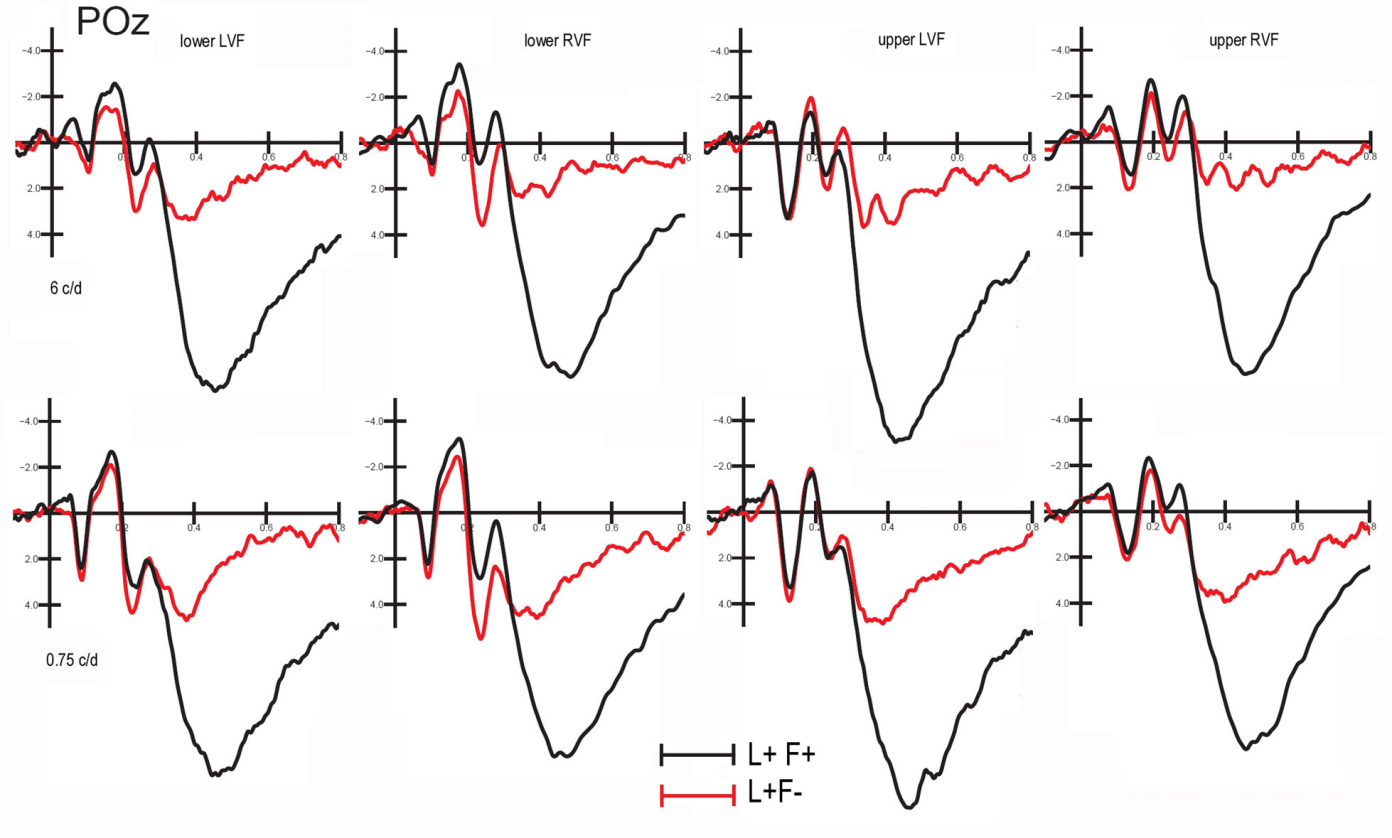
**Grand-average ERPs averaged across all subjects and recorded at POz mesial occipital site in response to targets (L+F+) and gratings of the irrelevant frequency falling at the relevant location (L+F-), separately for each quadrant of visual field and gratings spatial frequency**.

Selective attention to spatial and non-spatial stimulus properties strongly modulated both early and late cognitive components, namely a *selection negativity*, in the form of enhanced N1 and N2 components, and a central P3 component larger to attentionally relevant than irrelevant stimuli (see waveforms in Fig. 3, for an example). For the sake of brevity, only early-latency attentional effects will be discussed in this paper.

#### C1 (40-60 ms)

Statistical analyses performed on the mean area amplitude of C1 within the 40-60 ms time window showed that electrode site was significant (F[3,72] = 32.27, p < 0.00001) with larger negativities to mesial occipital rather than lateral occipital sites. Frequency relevance × location relevance × quadrant also reached significance (F[1,24] = 4.54, p < 0.01). Post-hoc comparisons indicated significant frequency-relevant effects at the attended locations (see waveforms in Fig. 3) especially for gratings falling in the LVF. The further interaction of frequency relevance × location relevance × hemisphere (F[1,24] = 6.40, p < 0.02) indicated a significant frequency-relevant effect with more negative C1 to F+ than F- gratings at both attended and unattended locations over right hemispheric sites, and at the attended location over the left hemisphere.

Simple effects on C40-60 recorded separately to gratings of 0.75 vs 6 c/deg were also analyzed. This ANOVA was justified by the much smaller (although significant) frequency relevance effect for the lowest spatial frequency, with a bandwidth far from the range of optimal frequency stimulation for the human visual system. It is conceivable that V1 neural cells might discriminate and preferentially select optimal frequencies earlier than non-preferred frequencies. The ANOVA recorded on C1 values to 6/deg between 40 and 60 ms revealed a significant frequency relevance effect *per se *(F[1,25] = 5.58, p < 0.03), with more negative C1 values to F+ than F- gratings. Location relevance × frequency relevance × hemisphere (F[1,25] = 4.57, p < 0.04) and electrode (F[3,75] = 31.22, p < 0.00001) were also significant factors. The ANOVA performed for 0.75 c/deg showed an effect of quadrant × frequency relevance × hemisphere (F[3,72] = 3.23, p < 0.03). Post-hoc comparisons showed that frequency relevance was significant for LVF but not RVF gratings.

The interaction quadrant × electrode (F[9,216] = 2.11, p < 0.03) comparison showed that N40-60 was more negative to upper than to lower gratings especially at mesial occipital sites.

**Figure 4:**
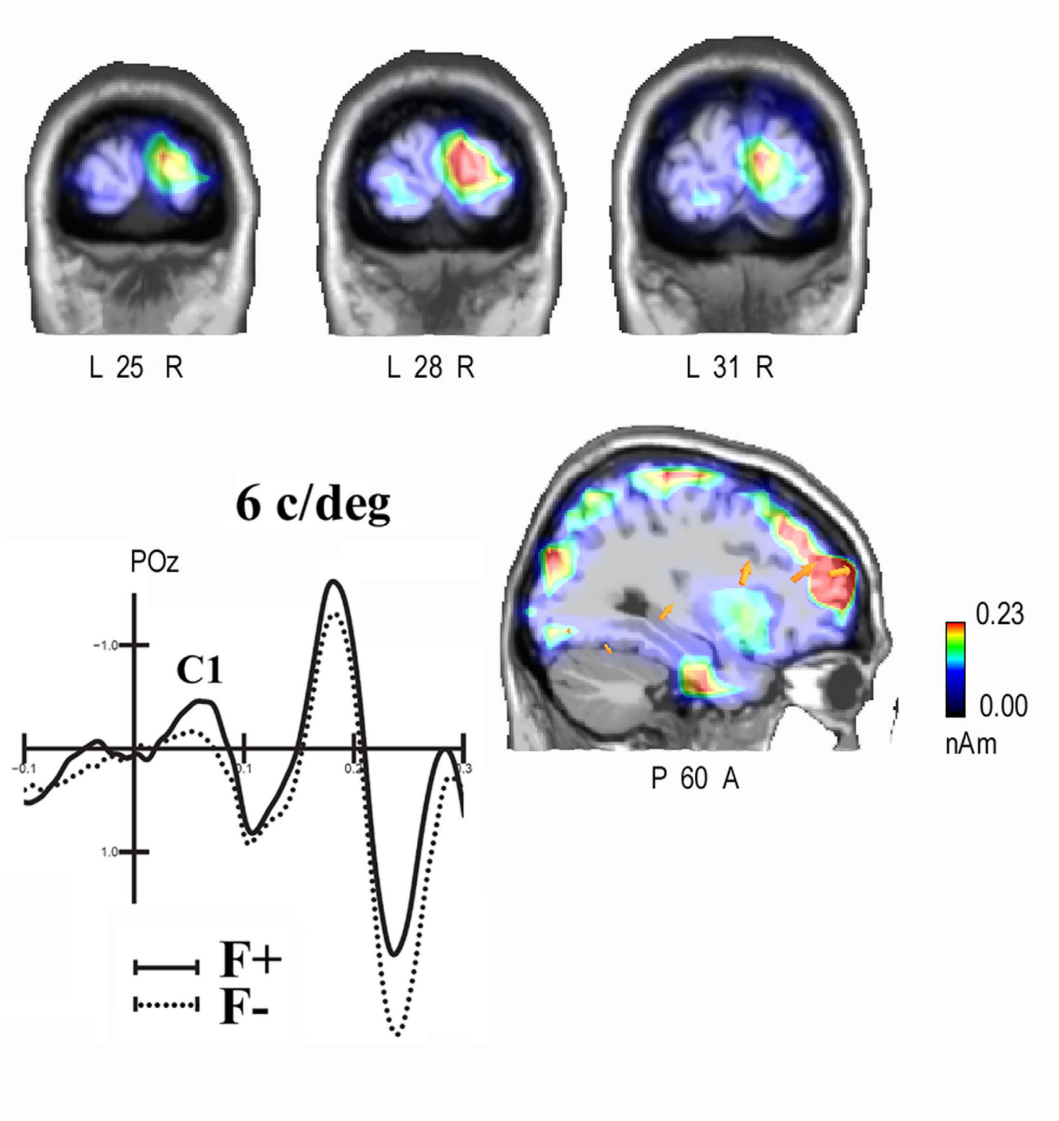
**swLORETA inverse solution performed on the difference wave F +/- F- in the time window 40-60 ms for 6/deg gratings**. It is visible a source of activation in the primary visual cortex (right cuneus, BA17). Yellow arrows indicate electromagnetic dipoles. In the lower left, grand-average ERPs to F+ and F- 6/deg gratings recorded at POz site.

In order to investigate the neural bases of the frequency-based attention effect, especially for the optimal frequency (6/deg), a difference wave was computed by subtracting ERPs to frequency-irrelevant (F-) from those to relevant (F+) 6/deg gratings regardless of location relevance. A LORETA inverse solution was performed on the F +/- F- difference wave in the time window 40-60 ms. The neural generators explaining the surface difference voltages are displayed as scans (see Fig. 4) and their Tailarach coordinates are listed in Table 1.

**Table 1:** List of active LORETA sources explaining the difference voltage: relevant - irrelevant spatial frequency (40-60 ms) for 6 c/deg gratings.

Magnit	T-x [mm]	T-y [mm]	T-z [mm]	Hem	Lobe	Area	BA
0.296	11.3	-98.5	2.1	Right	Occipital	Cuneus	17
0.312	31	-90.3	20.8	Right	Occipital	Middle Occipital	19
0.223	40.9	-76.2	-11.7	Right	Occipital	Fusiform	19
0.233	-18.5	-82.1	39.5	Left	Parietal	Precuneus	19
0.314	60.6	-55	-17.6	Right	Occipital	Fusiform	37
0.25	-8.5	-63.8	59	Left	Sup. Parietal	Parietal	7
0.52	70.5	-26.5	-0.6	Right	Middle Temporal	Temporal	21
0.708	60.6	3.3	20.5	Right	Frontal	Precentral	6
0.745	50.8	33.4	23.1	Right	Frontal	Middle Frontal	46
0.777	31	53.4	24.8	Right	Frontal	Superior Frontal	10
0.336	-28.5	53.4	24.8	Left	Frontal	Superior Frontal	10

Grid spacing = 5 mm, estimated SNR = 3.

The active sources included the right primary visual area (BA17), the lateral occipital area (BA19), the superior parietal lobule (BA7) and various dorsalateral prefrontal regions.

#### C1(60-80 ms)

**Figure 5:**
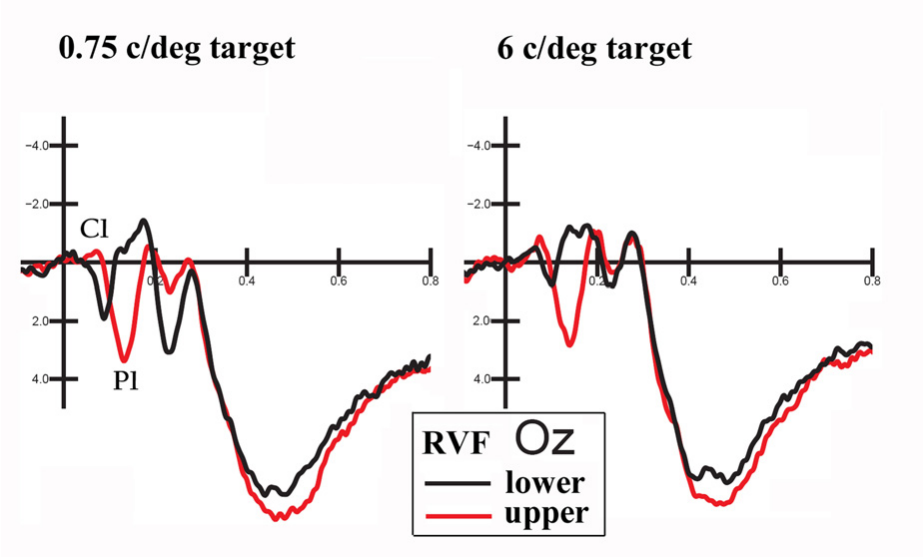
**ERPs recorded at OZ site in response to RVF upper and lower gratings as a function of spatial frequency**. The P/N80 (C1) inversion in polarity as a function of upper vs. lower quadrant of stimulation is clearly visible.

ANOVA performed on the mean amplitude values recorded in the time window 60-80 ms confirmed an inversion of P/N80 as a function of vertical meridian and spatial frequency, visible in Fig. 5.

Indeed, gratings of 6 c/deg elicited a larger N80 than gratings of 0.75 c/deg, irrespective of the attention condition, as suggested by the significance of spatial frequency (F[1,25] = 6.65, p < 0.02). Similarly, gratings presented in the upper quadrants elicited larger negativities (N80) than gratings presented in the lower quadrants, which elicited a P80 response instead, as confirmed by the significance of quadrants (F[1,25] = 12.9, p < 0.00001). Overall, C1 was more negative at mesial occipital than lateral occipital sites, as demonstrated by the electrode factor (F[3,75] = 18.04, p < 0.00001). Frequency relevance was significant *per se *(F[1,25] = 4.28, p < 0.049), indicating larger N80 to frequency-relevant than irrelevant gratings, as mostly evident in the waveforms shown in Fig. 4. The tendency toward significance of the frequency relevance × spatial frequency analysis suggested that attention effects were stronger for 6 c/deg than 0.75 c/deg gratings (F[1,25] = 3.81, p < 0.06). The interaction of location relevance × frequency relevance (F[1,25] = 6.22, p < 0.02) indicated a stronger frequency-relevance effect within the attended location; there was no difference between the F+L- and F-L- conditions.

The interaction of spatial frequency × quadrant (F[3,75] = 15.52, p < 0.00001) indicated a stronger effect of horizontal meridian (upper vs. lower) on the P/N80 morphology for low spatial frequency gratings, which elicited an N80 in response to upper stimuli (-0.64 μV) and a P80 in response to lower stimuli (0.46 μV), whereas 6 c/deg spatial frequency gratings elicited N80 responses of different amplitude to both upper (-0.39 μV) and lower quadrant gratings (-0.20 μV).

The interaction of quadrant × hemisphere (F[3,75] = 29.07, p < 0.00001) showed that while N80 was ispilateral to the stimulus field, P80 was contralateral to it, as confirmed by significant post-hoc comparisons.

**Figure 6:**
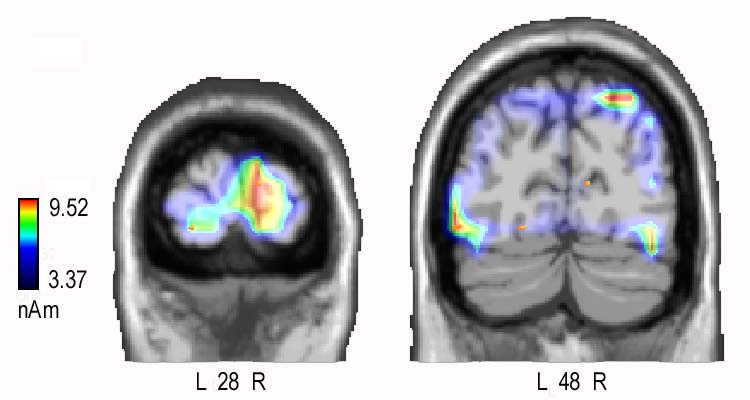
**swLORETA inverse solution performed on the difference wave F +/- F- in the time window 60-80 ms**. At this latency range a strong striate generator (BA17) was found, along with a bilateral extra-striate activation, visible at deeper coronal sections.

In order to investigate the neural bases of the frequency-based attention effect, a difference wave was computed by subtracting ERPs to frequency-irrelevant (F-) from ERPs to relevant (F+) gratings regardless of location relevance and stimulus spatial frequency. A LORETA inverse solution was performed on the F +/- F- difference wave in the time window 60-80 ms. The neural generators explaining the surface difference voltage are shown as scans (Fig. 6) and their Tailarach coordinates are listed in Table 2. The active sources included the right primary visual area (BA17), the lateral occipital area (BA18/19), the left parietal area (BA7/19) and various bilateral dorsalateral prefrontal, inferior frontal and superior frontal regions.

**Table 2:** List of active LORETA sources explaining the difference voltage: relevant - irrelevant spatial frequency (60-80 ms).

Magnit	T-x [mm]	T-y [mm]	T-z [mm]	Hem	Lobe	Area	BA
3.51	11.3	-98.5	2.1	Right	Occipital	Cuneus	17
3.02	-18.5	-96.5	-13.1	Left	Occipital	Lingual	18
3.52	-48.5	-76.2	-11.7	Left	Temporal	Fusiform	19
3.99	50.8	-66.1	-10.9	Right	Temporal	Fusiform	19
4.65	31	-73	49.2	Right	Sup. Parietal	Parietal	7
2.72	-58.5	-58.9	14.5	Left	Temporal	Superior temporal	22
4.00	60.6	-41.5	42.9	Right	Inf. Par	Parietal	40
3.75	-48.5	-32.4	52.7	Left	Inf. Par.	Parietal	40
5.82	-8.5	-15.8	63.3	Left	Frontal	Superior Frontal	6
7.94	60.6	13.3	21.4	Right	Frontal	Inferior Frontal	45
4.86	21.2	19.5	57.8	Right	Frontal	Superior Frontal	6
5.02	-48.5	25.3	4.4	Left	Frontal	Inferior Frontal	45
9.38	40.9	43.4	23.9	Right	Frontal	Middle Frontal	10
6.57	-38.5	43.4	23.9	Left	Frontal	Middle Frontal	10

Grid spacing = 5 mm, estimated SNR = 3.

#### C1(80-100 ms)

As in the previous temporal window, 6/deg gratings elicited more negative early responses than 0.75 c/deg gratings irrespective of attention condition (F[1,25] = 37.4, p < 0.00001). Again, stimuli falling in the upper quadrants elicited an N80 response while lower field stimuli elicited a P80 response as indicated by the significance of quadrant (F(3,75) = 11.84, p < 0.0001). The interaction of quadrant × location (F[3,75] = 3.12, p < 0.0376) indicated a larger effect of quadrant, with a P/N80 inversion of upper stimuli for location-relevant compared to irrelevant stimuli.

Spatial frequency relevance affected the amplitude of P/N80 *per se *(F[1,25] = 5.85, p < 0.02), with more negative responses to frequency-relevant than irrelevant stimuli.

N80 amplitude was greater at mesial occipital than lateral occipital sites as shown by the electrode factor (F[3,75] = 14.46, p < 0.00001).

The interaction of spatial frequency × quadrant (F[3,75] = 11.82, p < 0.00001) indicated that for each quadrant (except the left upper field), spatial frequency affected C1 amplitude with a more positive response to 0.75 and a more negative response to 6/deg gratings. As in the previous time window, the interaction of location relevance and frequency relevance was significant (F[1,25] = 4.72, p < 0.04), indicating a strong effect of spatial frequency relevance at the attended location. Furthermore, post-hoc comparisons showed a significant difference between L+F- (0.88 μV) and L-F- (0.58 μV).

The interaction of quadrant × hemisphere (F[3,75] = 27.69, p < 0.00001), indicated a larger C1 (more negative) in the ipsilateral hemisphere and a more positive P80 in the contralateral hemisphere.

#### P1(100-120 ms)

**Figure 7:**
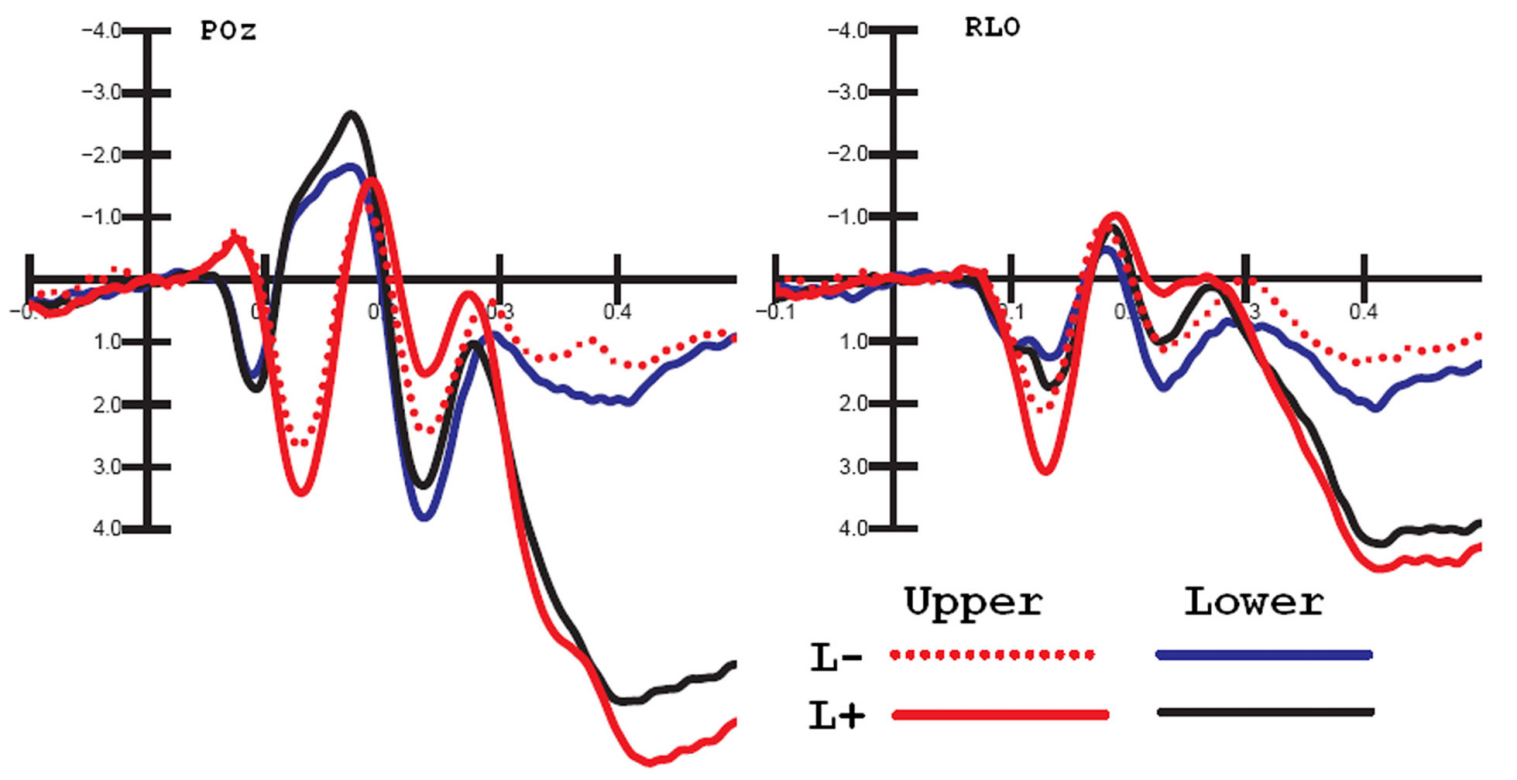
**Grand-average ERPs recorded to location-relevant and irrelevant gratings presented in the upper and lower hemi-spaces, independent of stimulus spatial frequency, and recorded at POz site and right lateral occipital site (RLO)**. Spatial attention effects were positive in nature.

ANOVA performed on the mean P1 amplitude values within the 100-120 ms time window showed the significance of location relevance *per se *(F[1,25] = 7.65 p < 0.01), with more positive responses to location relevant than irrelevant stimuli, as shown in the waveforms of Fig. 7. Overall, P1 was larger over the right hemisphere recording sites, as indicated by the hemisphere factor (F[1,25] = 4.68, p < 0.04). P1 was also larger at lateral occipital than mesial electrode sites (F[3,75] = 14.06, p < 0.00001).

The significant interaction of spatial frequency × quadrant (F[3,75] = 3.83, p < 0.02) indicated larger P1 responses to upper rather than lower stimuli (see Fig. 2), more evident for 6 c/deg gratings. The interaction of spatial frequency × location relevance (F[3,75] = 5.55, p < 0.03) showed significant space-based attention effects for both spatial frequencies, but stronger for 0.75 than 6 c/deg gratings. The interaction of location relevance × quadrant (F[3,75] = 4.39 p < 0.01) indicated robust and significant space-based attention effects at all locations, but particularly in the left upper field. Furthermore, the interaction of quadrant × hemisphere (F[3,75] = 9.2, p < 0.001) indicated larger P1 responses to contralateral rather than ipsilateral stimuli. As in the previous time windows, the interaction of location × frequency relevance (F[1,25] = 7.47 p < 0.01) was significant. P1 was larger to location relevant than irrelevant gratings, while frequency relevance at the attended location did not affect P1 amplitude in this latency range. The triple interaction of spatial frequency × frequency relevance × electrode site (F[3,75] = 3.13, p < 0.05) indicated significant frequency-based attention effects only for 6 c/deg gratings at the O1 and O2 electrode sites (p < 0.002).

#### P1 (120-140 ms)

P1 was strongly affected by location relevance in this time window with much larger P1 to attended locations, as in the previous time window (F[1,25] = 31.9, p < 0.00001). Also, the spatial frequency factor was significant (F[1,25] = 18.34 p < 0.005), with larger P1 responses to 0.75 c/deg than 6c/deg gratings, as indicated by the waveforms in Fig. 5. At this latency, P1 was larger over the right hemisphere (F[1,25] = 13.6, p < 0.0001), and over lateral/occipital than mesial occipital sites (F[3,75] = 15.5, p < 0.00002). It was also more positive in response to stimuli presented in the upper than in the lower quadrants (F[3,75] = 24.57, p < 0.00001), as exemplified in Fig. 5. The interaction of location relevance × frequency relevance (F[3,75] = 13.48, p < 0.0012) indicated a frequency relevance effect at both the attended and the unattended locations.

## Discussion and Conclusion

In this paradigm, gratings of different spatial frequencies were randomly presented in the upper and lower quadrants of the visual field in a task requiring conjoined/simultaneous attention to spatial location and spatial frequency. Visual evoked potentials (VEPs) showed the usual effects of spatial frequency and retinal position on the amplitude of sensory components, with larger N80 responses to upper stimuli and to higher spatial frequencies. P/N80 amplitude was greater at mesial occipito/parietal sites and inverted its polarity as a function of the above factors. As often reported, the later P120 response, greater at lateral occipital sites, had a larger amplitude to low spatial frequency gratings and showed the strongest spatial attention effects. Overall, these effects are rather canonical and are well documented in the literature [36,37]. Equally well known is the striate origin of the C1 component of VEPs, demonstrated by both electrophysiological and neuroimaging techniques [33,38-41].

The P/N80 inversion as a function of stimulus horizontal meridian and spatial frequency is highly consistent with previous electrophysiological literature [26,31,37,41,31,43,44].

ANOVA performed on the mean amplitude value of the C1 component in the first time window considered (40-60 ms) showed significant frequency-relevance effects at both attended and unattended locations at right hemispheric sites, and at the attended location at the left hemispheric sites. In addition, frequency-relevance effects at the attended location were larger for LVF gratings. This phenomenon might be interpreted in two ways. One possibility is that selective attention to spatial frequency exhibited the renowned hemispheric asymmetry for frequency processing, the right hemisphere being more efficient in processing the range of low (0.75 c/deg) than high (6 c/deg) spatial frequencies [45-47]. The other hypothesis is that the LVF/right hemisphere advantage might reflect an early low-level sensory bias for visual processing. Indeed, there is evidence of a similar LVF advantage for the processing of simple visual stimuli in simple RTS paradigms [48]. In any case, the matter deserves further investigation. The right hemispheric and LVF attention effects at the earliest stage of visual processing are strongly consistent with LORETA source reconstruction indicating an attentional effect for the relevant frequency (F+ vs. F-) in the right occipital cortex (BA17) in both C1 time windows (40-60 and 60-80 ms).

The early onset of the spatial frequency-based attention effect is compatible with the most recent findings on the timing of space-based attentional selection, e.g. [16]. In addition, the early (40-60 ms) emergence of robust 6 but not 0.75 c/deg frequency selection effects are compatible with recent findings [33] showing that at high contrast levels, the parvocellular system makes the biggest contribution to generating the C1 component starting at about 45 ms. Overall, evidence of stronger frequency relevance effects for high (6 c/deg) than low (0.75 c/deg) spatial frequency gratings has previously been reported in similar ERP attentional studies [22,25,26,31,32]. This inhomogeneity may be ascribed to the difference in contrast sensitivity across spatial frequency ranges, with 4-5/deg spatial frequency bandwidth being the optimal range for the human visual system [31,49,50]. In this light, it is conceivable that the earliest target/non-target effect might be observed in V1 for the frequency band eliciting the most optimal response among V1 neurons (6 rather than 0.75 c/deg). The preference for 6 over 0.75 c/deg gratings is also supported by behavioural data, showing faster RTs to the former stimuli.

The interaction between location relevance × frequency relevance, observable from the earliest sensory stages, suggesting stronger attentional selection effects at the attended location, is compatible with previous ERP literature [26,31,32] suggesting similar effects as early as 60 ms post-stimulus. The mechanism subserving this attention enhancement is probably related to the mechanism by which covert spatial attention increases contrast sensitivity via contrast gain, thus enhancing spatial resolution, described in neurophysiological and psychophysical studies [51,52].

These data strongly influence the existing assumptions and models of selective attention according to which the effects of attention on V1 activity take place not during the initial stimulus-related response (60-90 ms) but, instead, at longer latencies in the time range 150-250 ms, as a sort of re-entrant feedback [8,11,13].

The present data firmly establish that, indeed, as a result of task attentional relevance, visual cortex responsivity (including V1 activity) is cued to enhance/improve the processing of the attended spatial frequency, at both attended and unattended locations. While later (P1) frequency-relevance effects were stronger at the attended location (L+F+ vs. L+F-), the earliest frequency relevant effects, namely C1 modulation between 40 and 100 ms (see Table 1), exhibited strong frequency-relevance effects *per se *(F+ vs F-) (see Fig. 6). These data support the hypothesis that object-based selective attention processes might also be carried out at the earliest processing stage within the striate visual cortex, similarly to what was found for spatial attention most recently [16]. Indeed Kelly and coworkers employed a visuo-spatial task in which subjects were cued on each trial to direct attention toward 1 of 2 locations in anticipation of an imperative 6 c/deg Gabor stimulus and were required to detect a region of lower luminance appearing within the Gabor pattern 30% of the time at the cued location only. The data show a clear spatial attentional enhancement of the C1, beginning as early as its point of onset (57 ms), which inverted in polarity as a function of upper vs. lower hemispace. Source analysis of the attentional modulations pointed to generation in striate cortex.

It's interesting to note that, in our study, C1 attention effect did not invert in polarity as a function of quadrant of stimulation (as expected on the basis of P/N80 reversal to upper vs. lower stimuli). In fact, while location relevance did not affect much of the earliest sensory processing, and later on it enhanced the positivity of VEPs to gratings falling at the attended location, frequency relevance increased the negativity of both C1 and P1 responses regardless of quadrant of presentation. The presence of this attentional modulation, a sort of early *selection negativity *(SN) [22,25] that subsequently enhanced the amplitude of posterior N1 and N2 components (as clearly visible in Fig. 3 and 5), supports the hypothesis that C1 might index the activity of multiple generators beyond primary visual cortex.

These findings are paralleled by a number of electrophysiological data suggesting several sources for the early VEP based both on pathological [53] and control data. In addition, MEG findings [54,55] have demonstrated the involvement of V1, V2, V3, inferior and superior lateral occipital gyri and intraparietal sulcus in generating post-synaptic potentials in the 70-100 ms post-stimulus time window.

As for more anterior brain areas, in our study the frequency-based attention-related activation of BA6, BA45/46 and BA10 prefrontal areas was quite small in the early phase of C1 (below 0.7 nA of magnitude) but became much stronger and reliable (6-9 nA) in the next time window (60-80 ms): This pattern of results is consistent with the electrophysiological and SCD mapping data provided by Foxe and Simpson [56] showing an early activation of dorsolateral prefrontal cortex in the C1 range (as early as 80 ms) during a cued multisensory attention task. At this regard it should be considered the crucial role of the frontal lobe in spatial attention allocation, which may occur even before V1 response. It is for example known that the frontal eye field has neurons that discharge before visually guided saccades [57] thanks to corollary discharge signals coming from superior colliculus pathway and travelling through mediodorsal thalamus to the frontal eye fields, in the prefrontal cortex [58].Supporting evidence comes also from TMS studies showing the involvement of both frontal eye fields [59,60] and dorsolateral prefrontal cortex [61] in the early modulation of visual cortex during covert voluntary attention tasks.

Indeed, the direct role of the frontal lobe in modulating visual processing and particularly the V1 response has been demonstrated. For example it has been shown that single pulses of transcranial magnetic stimulation (sTMS) restricted locally to frontal cortical areas enhance visual perception of phosphenes and flashed alphabetical characters [62]. According to the authors, the anterior frontal lobe can gate information from primary visual cortical areas leading to enhanced perception through its powerful connections with the thalamic intralaminar system. It has been proposed that the frontal-lobe projections to the thalamic intralaminar nuclei can selectively enhance sensory processing by the primary cortical receiving area, thus giving rise to the early attentional modulation of V1.

It is quite interesting to consider at this regard that, in humans, activity of thalamocortical circuitry is reflected by gamma activity in the EEG [63,64] and indeed there is clear evidence of both beta and gamma synchronization around the time of C1, beginning around 50 ms. The oscillatory data suggest the possibility of long distance synchronization as an explanation of early V1 effects. Besides hard-wired anatomical pathways which could convey information to the occipital cortex at short latency, long distance synchronizing effects of attention on V1 neurons should be also be considered.

As for the potential limitations of this study it may be considered that fitting the total time period with ICA methods may have strengthened or weakened the conclusion of latency linked attention in V1. Further investigation will be able to shed some light on this matter.

In conclusion, the present data highlight the limitation of the current model of object-based visual selective attention in demonstrating that visual cortex responsivity (including V1 activity) is cued to enhance/improve the processing of attended objects at the earliest sensory level (C1).

## Authors' contributions

AMP and AZ conceived and designed the study, MDZ performed EEG acquisition and accomplished most of the data analyses. AMP interpreted the data and wrote the manuscript. All authors read and approved the final version of the manuscript.
